# Hypoxia Increases Connexin46 and Connexin43 Levels in KNS-42 Glioblastoma Cells

**DOI:** 10.3390/ijms27062851

**Published:** 2026-03-21

**Authors:** Peter J. Minogue, Eric C. Beyer, Viviana M. Berthoud

**Affiliations:** Department of Pediatrics, Biological Sciences Division, University of Chicago, Chicago, IL 60637, USA; pminogue@bsd.uchicago.edu (P.J.M.); ecbeyer@uchicago.edu (E.C.B.)

**Keywords:** connexin, glioblastoma, protein degradation, hypoxia, temozolomide, GBM

## Abstract

Glioblastoma multiforme is a devastating brain tumor that frequently progresses or recurs despite therapy. We used the glioblastoma-derived cell line, KNS-42, to study the response of the gap junction proteins, connexin46 and connexin43, to chemotherapeutic agents and to prolonged hypoxia to mimic conditions within the tumor microenvironment. Under standard culture conditions, KNS-42 cells have high levels of connexin43 and very low levels of connexin46. The cells that survived temozolomide treatment had increased connexin46 levels and decreased connexin43 levels. In contrast, prolonged hypoxia increased the levels of both connexins, the number of connexin immunopositive cells, and the intensity of the immunofluorescence signal per cell (which localized preferentially in the cytoplasm). Exposure to hypoxia for 12 days decreased the chymotrypsin-like proteasomal activity without altering connexin mRNA levels, suggesting that the changes in connexin levels result from reduced protein degradation. The increased connexin46 in temozolomide-resistant cells suggests that this connexin may have a role in chemotherapy resistance. The results also imply that changes in the microenvironment of glioblastomas (like hypoxia) can alter proteasomal activity and affect levels and subcellular distribution of connexin46 and connexin43. Finally, our data suggest that proteasomal inhibition may not be a good approach to glioblastoma therapy.

## 1. Introduction

The connexin proteins form gap junction channels, providing a pathway for direct communication between the cytoplasm of the cells participating in their formation. These channels are permeable to ions and molecules up to about 1 kDa. They also form “hemi-channels” at the plasma membrane that provide a pathway between intracellular compartments and the extracellular space. They have been implicated in the pathogenesis of cancer for many years (reviewed in [1,2]). Connexins may act as tumor suppressors or as tumor promoters, influencing cell proliferation, metastasis, and treatment resistance (reviewed in [2]). Whether a connexin acts as a tumor suppressor or promoter depends on connexin isoform and function, cancer type, and disease stage.

Several connexin isoforms have been implicated in cancer. Most studies have focused on connexin43 (Cx43) which is normally expressed in many cell types [3,4]. Cancer therapies that target Cx43 might have significant side effects by affecting the normal Cx43-expresing tissues. The expression of a different connexin, connexin46 (Cx46), has been reported in cells derived from various cancers, including breast cancer, retinoblastoma and glioblastoma [5,6,7]. Unlike the wide distribution of Cx43, Cx46 is found almost exclusively in the differentiating and mature fiber cells of the eye lens (reviewed in [8,9]). The fact that mutations that result in loss of Cx46 function are almost exclusively associated with cataracts in humans and mice (reviewed in [9,10]) implies that Cx46 has minimal functional importance outside the eye. Thus, Cx46 is a potential therapeutic target with fewer side effects. Moreover, studies of glioblastoma multiforme (GBM) reported that Cx46 is predominantly expressed in cancer stem cells [7], the population of cells which is likely responsible for the resistance and relapse of this cancer.

Gene expression data derived from many different cancer cells has been compiled in the DepMap portal [11]. We examined these expression data for all members of the connexin family (Table S1), and we found that cells derived from a variety of different cancers express Cx46 (together with varying levels of other connexins).

After screening several cell lines, we chose to focus on GBM. GBM is a devastating astrocytic brain tumor. Glioblastomas are very aggressive and have poor survival rates despite resection of the tumor and post-surgical adjuvant radio- and/or chemotherapy. All GBM cell lines express Cx43 and Cx46 mRNAs (except for a couple that do not have detectable Cx46; Table S1). Because normal astrocytes express Cx43, its presence in astrocytic tumors and glioblastoma-derived cells is not surprising [7,12,13,14,15,16]. However, the aberrant expression of Cx46 in GBM and its potential link to cancer “stemness” captured our attention. We sought to find a cell line that we could use as a model to study the potential roles of Cx46 (as compared to Cx43) in malignant astrocytes. The current study describes the initial characterization of connexin changes when these cells are exposed to chemotherapy or conditions that mimic tumor environmental conditions.

## 2. Results

### 2.1. KNS-42 Cells Make Gap Junction Plaques Containing Cx43 and/or Cx46

To identify an appropriate cell for studying the roles of Cx46 in cancer, we screened several available cells that seemed like reasonable candidates (including U87, KNS-42, and MCF7). All of these cells contained Cx43 and Cx46 mRNA (DepMap portal [11]; Table S1). While all of these cells contained abundant Cx43 protein, the levels of Cx46 protein were low (or not detectable) in all but KNS-42 cells. Therefore, we decided to use the KNS-42 cells for further studies. These cells were originally derived from a 16-year-old male patient with a grade IV malignant glioma (glioblastoma) who died one year after the appearance of symptoms [17]. According to the WHO CNS5 molecular classification of brain tumors, these cells fall into the glioblastoma, IDH-wildtype category [18]. KNS-42 cells are characterized by the H3.3 G34V mutation, and they also carry mutations of *TP53* and other genes.

**Figure 1:**
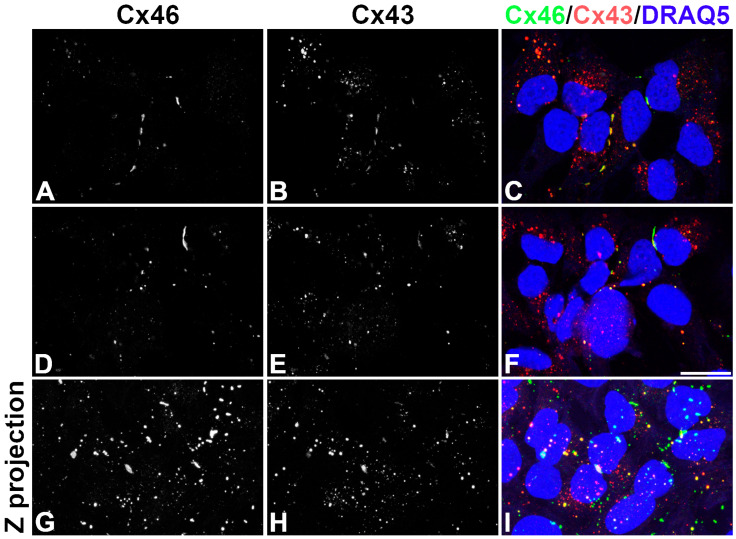
Both Cx43 and Cx46 are made in KNS-42 cells, but there is substantial variability of expression and of co-localization. (**A**–**F**) Single confocal plane images show the immunolocalization of Cx46 (**A**,**D**) and Cx43 (**B**,**E**) in two different groups of KNS-42 cells. (**G**–**I**) Z-Projection of confocal images in another group of cells showing the localization of immunoreactive Cx46 (**G**) and Cx43 (**H**). (**C**,**F**,**I**) Overlap of the Cx46 and Cx43 immunoreactivities (green and red, respectively) and DRAQ5-stained nuclei (blue). Superposition of the Cx46 and Cx43 immunoreactivities appears yellow. These images were selected to show that some cells contained both Cx46 and Cx43. Scale bar: 20 μm.

To test for the distribution of Cx46 in KNS-42 cells, we performed double-label immunofluorescence experiments. Because all GBM cells express Cx43, we also determined the distribution of Cx43 to assess how it compared with that of Cx46. Confocal analysis revealed that both connexins localized within the cytoplasm of some cells and to puncta at appositional plasma membranes, consistent with the formation of gap junction plaques (Figure 1). Not all cells expressed both connexins. While many cells expressed Cx43, Cx46 was detected only in a fraction of cells. In cells that expressed both connexins, there was substantial variation in the extent of their co-localization. Merged images showed overlap of both immunoreactivities, adjacent staining, or staining for only one of the connexins in different plaques (Figure 1).

### 2.2. Temozolomide-Resistant KNS-42 Cells Express Cx46

**Figure 2:**
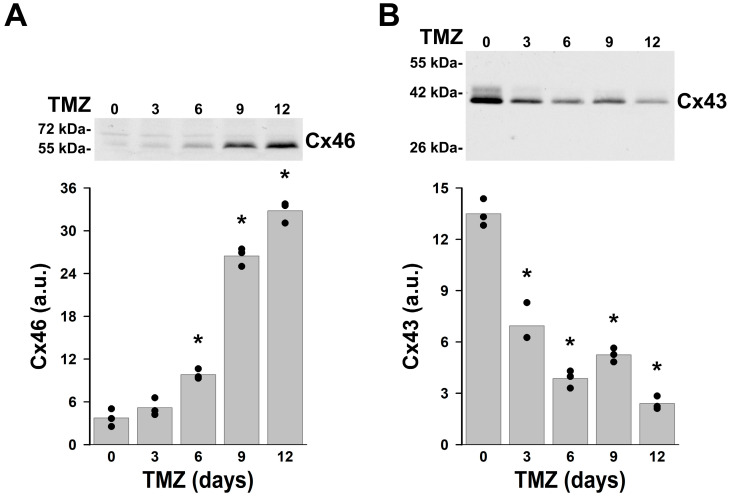
Treatment of KNS-42 cells with temozolomide alters the relative abundances of Cx43 and Cx46. (**A**,**B**) Immunoblots of Cx46 (**A**) and Cx43 (**B**) in total homogenates of KNS-42 cells incubated under normal conditions (0) or treated with 50 μM temozolomide (TMZ) for 3, 6, 9 or 12 days. The migration positions of the molecular mass markers are indicated on the left. Graphs depict the densitometric values of the bands detected in arbitrary units (a.u.). Black circles represent the densitometric value obtained in each of three independent experiments, and the gray bars represent the average of these values. Asterisks denote significant differences in Cx46 (**A**) or Cx43 (**B**) protein levels between untreated and TMZ-treated cells for the indicated number of days (*p* < 0.05).

Previous studies have suggested that Cx46 is present in glioblastoma and breast cancer “stem-like” cells and that its overexpression in breast cancer cells enhances “stem-like” characteristics [7,16,19]. Cancer stem cells exhibit increased resistance to many chemotherapeutic drugs, including the DNA alkylating agent, temozolomide. To test the effect of temozolomide on levels of Cx46 and Cx43 in KNS-42 cells, we treated these cells with temozolomide for several days and assessed connexin levels by immunoblotting. Untreated cells had very low levels of Cx46 and high levels of Cx43 (Figure 2). While many cells were killed by temozolomide, some cells survived. In the cell cultures that were treated with temozolomide, Cx46 levels increased and Cx43 decreased. After 12 days of temozolomide treatment, levels of Cx46 were 9.45 times as high as the levels detected in untreated cells whereas Cx43 levels were only 0.18 times as great as in untreated cells (Figure 2). The change in Cx46 levels correlated with an increase in the band with the highest electrophoretic mobility (~60 kDa) (Figure 2). These results support the existence of a Cx46-expressing subpopulation of KNS-42 cells that is temozolomide-resistant (a characteristic of glioma stem cells).

### 2.3. Hypoxia Increases Cx43 and Cx46 in KNS-42 Cells

**Figure 3:**
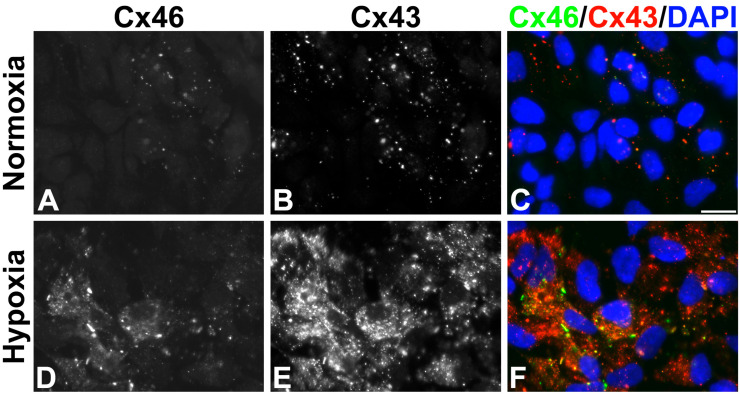
Hypoxic conditions increase Cx46 and Cx43 immunoreactivities in KNS-42 cells. (**A**–**F**) Photomicrographs show the distribution of immunoreactive Cx46 (**A**,**D**) and Cx43 (**B**,**E**) in KNS-42 cells cultured under normal conditions (**A**–**C**) or in 1% oxygen for 12 days (**D**–**F**). The overlap of the Cx46 (green) and Cx43 (red) signals with DAPI-stained nuclei (blue) is shown in (**C**,**F**). Superposition of the Cx46 and Cx43 immunoreactive signals appears yellow. Scale bar: 20 μm.

Cells within the interior of a solid tumor are exposed to a hypoxic environment. To test the effect of hypoxia on Cx43 and Cx46 in KNS-42 cells, the cells were cultured under hypoxic (1% O_2_) or normoxic (~21% O_2_) conditions. Cells subjected to prolonged hypoxia showed gap junctions containing Cx46 and/or Cx43 and a strikingly pronounced increase in cytoplasmic connexin immunoreactivity (Figure 3).

**Figure 4:**
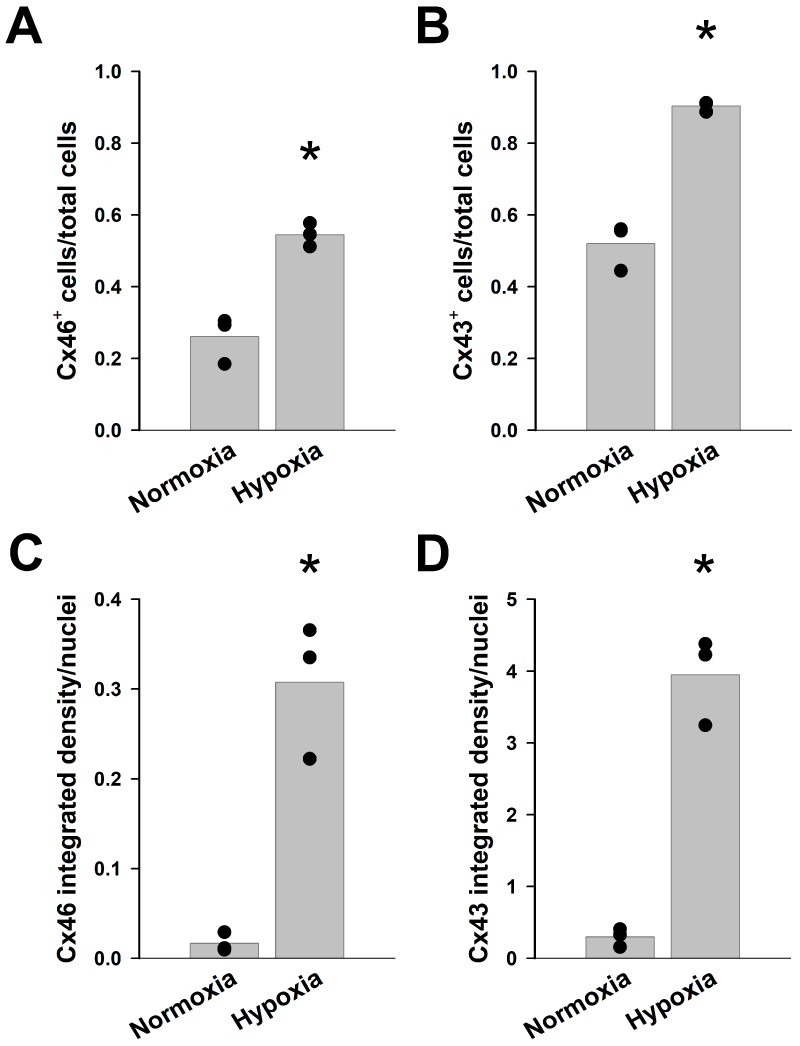
Chronic hypoxia increases both the number of KNS-42 cells showing Cx46 or Cx43 immunoreactivity and the integrated density of fluorescence per cell. (**A**,**B**) Graphs show the proportion of Cx46-positive (**A**) and Cx43-positive (**B**) cells with respect to the total number of cells in KNS-42 cells cultured under normal conditions (Normoxia) or in 1% oxygen for 12 days (Hypoxia) in three independent experiments (black circles). The bars represent the averages of the values obtained in each experiment. (**C**,**D**) Graphs show the immunoreactive integrated densities per nuclei for Cx46 (**C**) and Cx43 (**D**) in KNS-42 cells incubated under normoxic or chronic hypoxic conditions obtained in each of three independent experiments (black circles) and their averages (bars). Significant differences between the values obtained under normoxia and hypoxia (*p* < 0.05) are indicated by asterisks.

Under normal conditions, about a quarter of the cells showed Cx46 immunoreactivity, while about half of the cells were immunopositive for Cx43 as detected by immunofluorescence (Figure 4). The proportions of cells expressing either of these connexins increased substantially when cells were cultured in hypoxic conditions for 12 days. The fraction of cells containing immunoreactive Cx46 increased from 26% to 54.5%, and that containing Cx43 increased from 52% to 90.3% (Figure 3 and Figure 4A,B). In addition, the intensity of immunofluorescent staining for both connexins was much stronger under hypoxic conditions than in normoxia. We quantified the immunofluorescent signal as the integrated density per nucleus; under hypoxic conditions, the Cx46 signal was 18.6 times as high as the value in normoxia and the Cx43 signal was 13.3 times as high as its normoxic value (Figure 3 and Figure 4C,D).

We also quantified the numbers of cells showing staining for either or both connexins. After chronic hypoxia, a larger percentage of the cell population was immunopositive for both connexins (19% in normoxia vs. 52% in hypoxia). This change was associated with a concomitant decrease in the proportion of cells showing only Cx46 immunoreactivity (from 7.2% to 2.2%) without a significant change in the fraction of cells showing only Cx43 immunoreactivity (3.3% in normoxia vs. 3.8% in hypoxia) (Figure S1).

**Figure 5:**
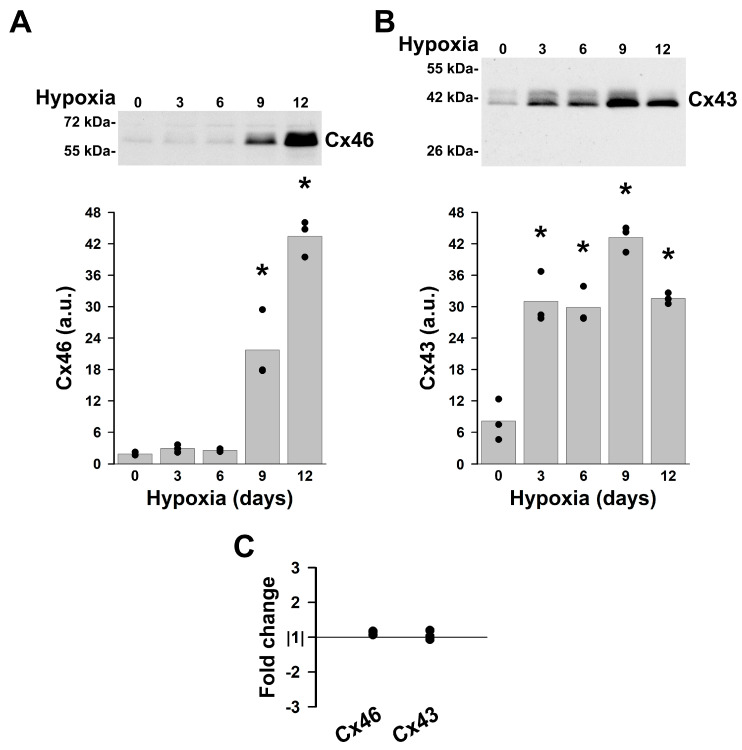
Chronic hypoxia increases Cx46 and Cx43 protein but not mRNA levels in KNS-42 cells. (**A**,**B**) Immunoblots of Cx46 (**A**) and Cx43 (**B**) in total homogenates of KNS-42 cells incubated under normal conditions (0) or in 1% hypoxia for the indicated number of days. The migration positions of the molecular mass markers are indicated on the left. Graphs show the densitometric values of the bands obtained after quantification of the bands detected in three independent experiments (black symbols, one for each experiment) in arbitrary units (a.u.). The bars represent the average of the densitometric values obtained in each experiment. Asterisks denote significant differences in Cx46 (**A**) or Cx43 (**B**) protein levels between cells subjected to hypoxia for the indicated number of days and cells incubated in normoxia (*p* < 0.05). (**C**) Graph shows the fold change in the transcript levels for Cx46 and Cx43 in KNS-42 cells incubated under 1% oxygen for 12 days relative to cells incubated under normal conditions as determined by Reverse Transcription Real-Time PCR. The values obtained in each of three independent experiments are shown in black circles. The value obtained in KNS-42 cells incubated in normoxic conditions was considered as |1|.

To quantify changes in levels of Cx46 and Cx43 induced by hypoxia and to determine the temporal course of these changes, KNS-42 cells were incubated under normal conditions or in 1% O_2_ for 3, 6, 9 or 12 days. Immunoblots showed that Cx43 levels increased within three days of hypoxia. After 3, 6, or 12 days in hypoxia, Cx43 levels were 4.3 to 4.5 times as high as in cells maintained in normoxia. Cx43 levels had a transient peak at 9 days of hypoxia, reaching 6.1 times the values under normoxic conditions. In contrast, no changes in Cx46 levels were detected after 3 or 6 days in hypoxia. After 9 days of culture in hypoxic conditions, levels of Cx46 were 12 times the values in cells cultured under normoxic conditions. Cx46 levels continued increasing to reach 23.6 times the normoxic values after 12 days of hypoxia (Figure 5A,B). The changes in connexin levels were associated with alterations in the immunoblot pattern. While the Cx43 band with the fastest electrophoretic mobility (41 kDa) became the predominant band after 12 days in hypoxia, almost all Cx46 was detected as two broad bands centered at ~60 and 64 kDa (Figure 5A,B).

To test whether the changes in connexin levels resulted from changes in expression, levels of Cx46 and Cx43 mRNA were determined after 12 days of hypoxia. The levels of these transcripts were similar to those of cells cultured under normoxic conditions (Figure 5C). This suggests that the changes in protein levels were not caused by increased connexin gene expression.

### 2.4. Chronic Hypoxia Decreases Proteasomal Activity in KNS-42 Cells

**Figure 6:**
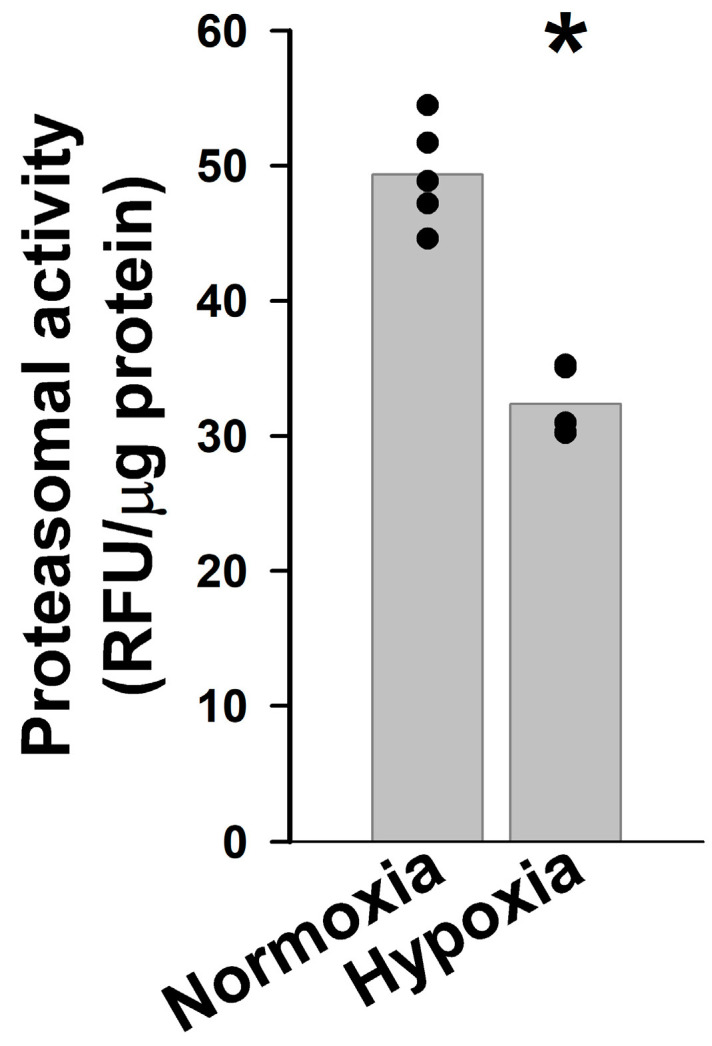
Proteasomal activity is decreased in KNS-42 cells by chronic hypoxia. Graph shows the activity of the proteasome in KNS-42 cells incubated at normal oxygen levels (Normoxia) or in 1% oxygen for 12 days (Hypoxia) in relative fluorescence units per μg of protein in five independent experiments (black circles). The gray bars represent the average of these values. The asterisk indicates a significant difference in activity between hypoxic and normoxic conditions (*p* < 0.05).

The increased levels of both Cx43 and Cx46 in cells cultured for 12 days under hypoxia when no changes in the levels of their mRNAs were detected suggested that hypoxia was affecting the degradation of these connexins. Since hypoxia induced by 1% O_2_ severely reduces the activity of the 26S proteasome leading to decreased degradation of some proteins in other cell types [20,21], we assessed the activity of this degradation pathway in KNS-42 cells cultured under normal (normoxic) or hypoxic (1% O_2_) conditions for 12 days. The chymotrypsin-like activity of the proteasome was significantly decreased in cells cultured for 12 days in hypoxic conditions. These cell cultures contained only 65.7% of the proteasomal activity present in cultures incubated in normal conditions (Figure 6).

## 3. Discussion

In this study, we demonstrated that KNS-42 glioblastoma cells contained detectable levels of both Cx43 and Cx46 proteins, although the fraction of cells containing immunodetectable Cx43 was much greater than that containing Cx46 under baseline conditions. Distinct gap junction plaques containing Cx43 and/or Cx46 were detected in some KNS-42 cells in normal conditions as well as under hypoxia. The detection of Cx43 was anticipated, because this connexin is expressed in normal astrocytes and in many different glioma cells [7,12,13,14,15,16]. Our observations demonstrating the presence of Cx46 extend previous studies that detected Cx46 mRNA and/or protein in several different kinds of malignant cells, including breast cancer, retinoblastoma and glioma stem-like cells [5,6,7,11,16].

The differential effect of temozolomide on Cx46 and on Cx43 likely arises from its mechanism of action. Temozolomide is a DNA alkylating agent that causes cell death and is commonly used as an adjuvant therapy to treat GBM after surgical resection. Indeed, while many KNS-42 cells did not survive treatment with temozolomide, a few of them were resistant. We observed a very large and significant increase in Cx46 levels concomitant with a major decrease in Cx43 levels in the KNS-42 cells that survived the temozolomide treatment. These findings imply a correlation between expression of Cx46 in KNS-42 cells and the ability of glioma stem cells to resist temozolomide treatment. These results are consistent with the findings in other glioblastoma cells reporting that stem-like cells (identified based on tumor initiation or expression of markers like CD133 and SOX2) express Cx46 [7,16]. Thus, our results suggest that tumor recurrence after resection and temozolomide treatment may be due to the survival of Cx46-expressing cells. These results also imply that Cx43 is not prevalent in the cancer stem-like cell population, since we observed decreased levels of Cx43 in the KNS-42 cells that survived temozolomide treatment. However, Cx43 expression has been reported by other scientists to be essential for survival of patient-derived glioblastoma cancer stem cells [22].

KNS-42 cells cultured under hypoxic conditions showed large increases in the levels of both Cx43 and Cx46. The increases in connexin protein levels detected under chronic hypoxia were due to an increased proportion of cells expressing either or both connexins and a very large increase in the immunoreactive signal per cell. While the hypoxic cells contained some Cx43 or Cx46 that localized to gap junction plaques at appositional membranes, much of the connexin localized intracellularly (presumably in the biosynthetic or degradative pathways). Thus, the majority of this connexin is not supporting known functional roles of plasma membrane-localized connexins. However, recent connexin-targeting approaches to glioma therapy that disrupt interactions of Cx43 with cellular proteins (e.g., Src and tubulins) have implicated cytoplasmic connexin domains in the mechanism of the disease. Treatment with peptides that target the Src-Cx43 or tubulin-Cx43 interactions (which occur in the intracellular carboxyl terminus of Cx43) decreased viability, migration, and invasion of glioma stem cells, limited xenograft tumor growth *in vivo*, and increased survival of mice [23,24,25,26]. In addition, cytoplasmic Cx43 modulates c-Myc expression in glioblastoma cells through a mechanism that involves participation of WNK lysine-deficient protein kinase 1, WNK1, which is important for maintaining glioblastoma stem cell characteristics (e.g., self-renewal, proliferation, survival, and tumor initiation) [22].

Increases in Cx46 levels in hypoxic conditions have been reported in normal lens cells and in cancer cells [5]. Because of this, it has been suggested that Cx46 provides cells with resistance to hypoxia [5]. This may be applicable to KNS-42 cells. However, chronic hypoxia not only increased levels of Cx46, but also increased levels of Cx43 in KNS-42 cells. In other cells (e.g., lens cells, Y79 retinoblastoma cells and breast cancer cells), hypoxia led to increased Cx46 levels and decreased Cx43 levels, knockdown of Cx46 resulted in increased Cx43 levels, and knockdown of Cx43 increased Cx46 levels [6,27]. These results led the authors to suggest that there is a reciprocal relationship between Cx43 and Cx46 expression. However, our results do not show such a relationship in the KNS-42 cell cultures subjected to prolonged hypoxia.

Several factors may contribute to increasing cellular protein levels, including increased synthesis and decreased degradation. The lack of significant changes in Cx46 and Cx43 mRNA levels in KNS-42 cells subjected to hypoxia for 12 days (when levels of Cx46 and Cx43 protein were markedly increased) excludes a change in gene transcription. Thus, the change in protein levels is likely to result from reduced degradation. In the hypoxic cells, we detected a significant decrease in proteasomal activity. The proteasome has been implicated in the degradation of both cytoplasmic and plasma membrane-localized connexins, including Cx43 and Cx46 ([28,29,30,31,32]; reviewed in [33,34]). Because of this correlation, we infer that the significant increase in both Cx46 and Cx43 detected in KNS-42 cells after exposure to chronic hypoxia likely results from a prolonged decrease in proteasomal activity. Although not tested, there could also be reduced lysosome-dependent degradation in the hypoxic cells. Indeed, hypoxia-induced alterations in cellular proteostasis could contribute to the stabilization of multiple proteins, with connexins representing one example of this broader effect. In support of our interpretation, proteasome inhibitors have been found to be relatively ineffective at increasing survival of patients with GBM [35,36].

Connexins are subject to co- and post-translational modification (reviewed in [37]). Modification of connexins by phosphorylation has been involved in their localization and stabilization at the plasma membrane forming gap junction plaques [38,39]. Both Cx46 and Cx43 are phosphoproteins, and several of their phosphoforms decrease the electrophoretic mobility of the protein (reviewed in [37,40]). Most connexins have half-lives in the order of a few hours (reviewed in [41]). The half-life of Cx43 varies between 1.3 and 3.3 h depending on the experimental system. Two kinetic pools of chicken Cx46 (aka Cx56) containing phosphoforms have been described; the pool with the slower electrophoretic mobilities has a half-life of days, while the other pool has a similar half-life to Cx43 (i.e., a few hours) [42]. The alterations in the immunoblot pattern of Cx46 in KNS-42 cells subjected to chronic hypoxia imply increased and heterogeneous modification of Cx46 (e.g., phosphorylation, ubiquitination, etc.). This treatment decreased phosphorylation of Cx43, especially in the phosphoform responsible for stabilization of gap junction plaques. In agreement with this interpretation, most of the immunoreactive Cx43 detected in the hypoxia-subjected cells localized in the cytoplasm.

A general feature of stem cells/cancer initiating cells is that they have lower proteasomal activity than the differentiated cells, as reported for the murine ES cell line E14.1 and several human and murine glioma and breast cancer cell lines (e.g., gliomas: human U87 MG and murine GL261 and 67NR; breast cancer: murine 67NR and human U343 and MCF-7) [43,44]. Interestingly, long-term (chronic) hypoxia of hepatocellular carcinoma cells increases the proportion of cells that behave like stem cells (i.e., they have stem cell surface markers and augmented tumorigenicity *in vivo*); these cells also have low proteasomal activity [45]. This seems to be the case for KNS-42 cells incubated for long periods under hypoxic conditions.

Temozolomide and prolonged hypoxia increased Cx46 levels, whereas these treatments had differing effects on the abundance of Cx43. A possible explanation is that there is a progression of changes in connexin levels between the cancer stem cells and the cells that make up the bulk of the tumor as suggested graphically by Hitomi et al. [7]. The cells exposed to hypoxia may represent an intermediate stage (with substantial levels of both Cx43 and Cx46). Changes in the microenvironment of solid tumors (like hypoxia) reduce proteasomal activity resulting in increases in protein levels of Cx46 and Cx43 (and likely many other proteins). This is consistent with observations that stem and cancer initiating cells have decreased proteasomal activity compared to differentiated cells [43,44,45] and that levels of Cx43 in tumors are lower than in normal non-tumoral tissue [15,46,47]. It would make sense that under hypoxic conditions, cells would try to compensate and support each other by increasing levels of connexins in an attempt to maximize intercellular communication. Since Cx43 has been shown to have a channel-independent function in cell migration [48], the increase in Cx43 levels in KNS-42 cells and its preferential localization in the cytoplasm may increase migratory capabilities of the cells and contribute to tumor cell migration and spread as suggested by Smyth et al. after targeting the tubulin-Cx43 interaction in other glioblastoma models [26]. The interactions of cytoplasmic connexin or connexin domains with tyrosine kinases or microtubules may alter the malignant phenotype [23,26]. In contrast, the cells that survive prolonged exposure to cytotoxic levels of temozolomide represent the stem-like cells (containing greatly increased levels of Cx46 and reduced levels of Cx43). These cells have the potential to “differentiate” to form the “intermediate” cells and then tumors containing predominantly cells with low levels of Cx43 and very low Cx46. Our results suggest that a therapy targeting both Cx46 and Cx43 in combination with a conventional cytotoxic chemotherapy drug like temozolomide might be more effective for the treatment of GBM.

### Limitations of the Study

Our experiments were performed using a single GBM cell line, which may raise concerns about the generality of the findings. However, (a) Cx46 is expressed in almost all the GBM cell lines compiled in the DepMap portal [11]; (b) in contrast to Cx43, which is prevalent in non-stem cancer cells, Cx46 is prevalent in cancer stem cells [7]; (c) Cx46 gap junction-mediated intercellular communication has been reported to be crucial for GBM cancer stem cells [7]; (d) treatment with gap junction inhibitors or clofazimine (which preferentially inhibits Cx46 gap junction channels) or Cx46 knockdown decreases GBM stem cell proliferation, self-renewal and tumor volume [7,49]; (e) Cx46 is upregulated in MCF-7 cancer cells and in human breast cancer [5]; (f) overexpression of Cx46GFP in breast cancer cells increases cancer stem cell characteristics [19]; (g) knockdown of Cx46 in xenograft tumors of Y79 retinoblastoma cells and MCF-7 cells decrease tumor volume/growth [5,6]; and (h) several types of stem cells have decreased proteasome activity [43,44,45]. Thus, several studies on different types of cancers implicate a crucial role for Cx46 in cell proliferation, self-renewal and ability to form spheres in tissue culture and xenografts *in vivo* (all characteristics of stem-like cells). All of these data give significant support to the interpretation of the data presented in this study.

## 4. Materials and Methods

### 4.1. Reagents and Antibodies

Temozolomide was obtained from Millipore Sigma (Burlington, MA, USA). N-Succinyl-Leu-Leu-Val-Tyr-7-amino-4-methylcoumarin (Suc-LLVY-AMC) was procured from Enzo Life Sciences (Farmingdale, NY, USA). cOmplete EDTA-free protease inhibitor mixture was obtained from Roche Applied Science (Indianapolis, IN, USA). Cytiva Amersham™ ECL™ Western Blotting Detection Reagents, DRAQ5™ Fluorescent Probe Solution (1,5-bis{[2-(di-methylamino) ethyl]amino}-4,8-dihydroxyanthracene-9,10-dione, 5 mM), DAPI (4′,6-diamidino-2-phenylindole dihydrochloride) were acquired from ThermoFisher Scientific (Waltham, MA, USA). Protein Assay Dye Reagent Concentrate was purchased from Bio-Rad (Hercules, CA, USA). All other chemicals were obtained from Sigma-Aldrich (St. Louis, MO, USA) or ThermoFisher Scientific.

The affinity purified rabbit polyclonal anti-Cx46 antibodies were generated in our laboratory and have been characterized previously [50]. Mouse monoclonal anti-Cx43 antibody (MAB3067) and polyclonal anti-Cx43 antibody (C6219) were acquired from Millipore Sigma. Alexa 488-conjugated AffiniPure F(ab′)_2_ fragment goat anti-rabbit IgG (111-546-144; lot 169878), Cy3-conjugated AffiniPure F(ab′)_2_ fragment goat anti-mouse IgG (115-166-146; lot 166579), and HRP-conjugated goat AffiniPure anti-rabbit IgG (H+L) (111-035-144; lot 157038) antibodies were obtained from Jackson ImmunoResearch (West Grove, PA, USA).

### 4.2. Tissue Culture

KNS-42 cells, an established human glioblastoma, IDH-wildtype cell line, were a kind gift from Dr. Andrea Piunti (University of Chicago). These cells were originally obtained from the Japanese Collection of Bioresources [51] where they were authenticated by STR profiling. Lack of mycoplasma contamination was verified using the Universal Mycoplasma Detection Kit from ATCC (Manassas, VA, USA). Cells were cultured in Dulbecco’s Modified Eagle Medium (DMEM, ThermoFisher Scientific) containing 10% fetal bovine serum (GeminiBio, Sacramento, CA, USA), 1× Minimum Essential Medium (MEM) non-essential amino acids (ThermoFisher Scientific), 10 units/mL penicillin and 10 μg/mL streptomycin (ThermoFisher Scientific). For normal conditions (or normoxia), cells were grown at 37 °C in a humidified 5% CO_2_/95% air tissue culture incubator. For hypoxic conditions, cells were continuously exposed to 5% CO_2_/1% O_2_ in a humidified hypoxia chamber at 37 °C. For long term hypoxia experiments, the tissue culture dishes in which cells were growing were wrapped in parafilm, removed from the chamber, and checked for confluence. When confluent, cells were split within the hypoxia chamber. Media for feeding and splitting cells were equilibrated to hypoxic conditions for at least 24 h.

### 4.3. Protein Quantification

Protein concentrations were determined using the Bio-Rad Protein Assay Dye Reagent Concentrate based on the method of Bradford [52].

### 4.4. Immunoblotting

Total homogenates of KNS-42 cells grown in 100-mm dishes under normal (normoxic) conditions, under hypoxia, or treated with temozolomide were prepared in PBS (pH 7.4) containing 4 mM EDTA, 2 mM PMSF, and cOmplete EDTA-free protease inhibitor mixture. One hundred μg (Cx46) or 2 μg (Cx43) of protein from each homogenate were loaded per lane on SDS-containing polyacrylamide gels and subjected to immunoblotting as previously performed [53] using anti-Cx46 and anti-Cx43 antibodies. Binding of secondary antibodies was detected using Cytiva Amersham ECL Western Blotting Detection Reagents (ThermoFisher Scientific). The bands detected were quantified by densitometry using Adobe Photoshop CS3 (Adobe Systems Inc., San Jose, CA, USA) in at least 3 independent experiments by drawing a rectangular box that encompassed each band. The box size was kept constant for all lanes in an individual blot. The same size box was used to obtain a background value in each lane and correct the integrated band density. The results are reported in arbitrary units. Graphs were prepared using SigmaPlot version 10.0 (Systat Software, Palo Alto, CA, USA).

### 4.5. Immunofluorescence

KNS-42 cells were grown on glass coverslips. They were fixed in 4% paraformaldehyde in PBS (pH 7.4) for 15 min at room temperature and subjected to immunofluorescence as previously described [54] using anti-Cx46 and anti-Cx43 antibodies. Nuclei were stained with DAPI or DRAQ5. Coverslips were mounted using 4% n-propylgallate in PBS/glycerol (1:4). Specimens were observed with a Leica HC PL APO 63× (numerical aperture 1.4) CS2 oil immersion objective using a Leica SP8 laser scanning confocal microscope (Leica Microsystems, Deerfield, IL, USA) or with a Zeiss Plan Apochromat ×40 (numerical aperture 1.0) objective in an Axioplan 2 microscope (Carl Zeiss Inc., White Plains, NY, USA) equipped with a mercury lamp and a Zeiss AxioCam digital camera. Pictures were taken from random visual fields. The results were quantified by two investigators in an independent and unbiased manner. The number of cells immunoreactive for only Cx46 or Cx43 and those immunoreactive for both connexins was quantified in 7 images from each of 3 independent experiments using ImageJ 1.54j [55]. These values were used to calculate the proportion of cells immunopositive for one or both connexins vs. the total number of cells and the average of these measurements for each of these experiments. The integrated density (mean intensity × area) of immunoreactive Cx46 and Cx43 per nuclei was calculated in each of 3 independent experiments as the average of values obtained by analyzing twenty-five images from each of these experiments using Fiji (ImageJ 1.54p) [56]. The data are presented as graphs in which symbols represent the average value of each independent experiment, and the bar represents the overall average of the 3 experiments. Representative images were used to illustrate the results. Figures were prepared using Adobe Photoshop 2025 (Adobe Systems). All panels in Figure 1 were equally brightened. Equal contrast adjustment was applied to each panel in Figure 3.

### 4.6. Reverse Transcription Real-Time PCR

KNS-42 cells were grown in 100-mm tissue culture dishes and incubated under normoxic conditions or for 12 days under hypoxic conditions. Then, cells were rinsed twice with PBS, pH 7.4, scraped from the dish directly in QIAzol Lysis Reagent (Qiagen, Germantown, MD, USA) and their RNAs were isolated using the Qiagen miRNeasy Mini Kit (Qiagen). Then, the samples were stored at −20 °C until processed for reverse transcription real-time PCR (RT-qPCR) as described previously [53] using primers for human Cx46 (sense, CGGACCTACGTCTTCAACATC and antisense, GAGATGAAGCAGTCCACCG), human Cx43 (sense, GGAGTTCAATCACTTGGCGT and antisense, ACACCTTCCCTCCAGCAGTT), and for human β_2_-microglobulin (B2M) (sense, TGCTGTCTCCATGTTTGATGTATCT and antisense, TCTCTGCTCCCCACCTCTAAGT). The transcript for B2M was used as a housekeeping gene to normalize levels of mRNA and allow comparisons between the samples.

### 4.7. Proteasomal Activity Assay

KNS-42 cells were grown on 100-mm tissue culture dishes under normoxic conditions or for 12 days under hypoxic conditions. Cell lysates were prepared, and the proteasomal chymotrypsin-like activity was determined using the fluorogenic substrate Suc-LLVY-AMC according to a previously published method [57]. Samples were read on a SpectraMax iD5 (Molecular Devices, San Jose, CA, USA).

### 4.8. Statistics

Normal distribution of the data was confirmed using the Shapiro–Wilk test. Then, the data were analyzed for statistical significance using 2-tailed unpaired Student’s *t*-test; a *p* value < 0.05 was considered significant.

## Data Availability

The original contributions presented in this study are included in the article/Supplementary Materials. Further inquiries can be directed to the corresponding author.

## Supplementary Materials

The following supporting information can be downloaded at: https://www.mdpi.com/article/10.3390/ijms27062851/s1.

## Abbreviations

The following abbreviations are used in this manuscript:

Cx	Connexin
DAPI	4′,6-diamidino-2-phenylindole dihydrochloride
DRAQ5	1,5-bis{[2-(di-methylamino) ethyl]amino}-4,8-dihydroxyanthracene-9,10-dione
GBM	Glioblastoma multiforme
RT-qPCR	reverse transcription real-time PCR
