# Supplementary material for: Hypoxia Increases Connexin46 and Connexin43 Levels in KNS-42 Glioblastoma Cells
Source: Int J Mol Sci. 2026 Mar 21;27(6):2851. doi: 10.3390/ijms27062851 (PMC13026484; doi:10.3390/ijms27062851)
Supplement: Supplementary file 1 [file ijms-27-02851-s001.zip › ijms-4181712-supplementary/IJMS-4181712-R1 Minogue et al Supplementary Material/IJMS-4181712-R1 Minogue et al Figure S1.pdf]

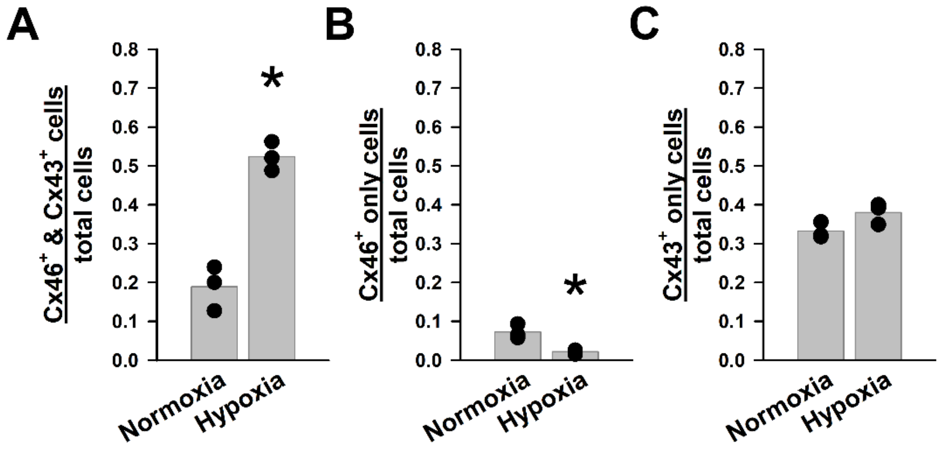

Figure S1. Hypoxia increases the proportion of cells that express both Cx46 and Cx43 and decreases the proportion of cells that express only Cx46. (A–C) Graphs show the proportion of KNS-42 cells that were immunoreactive for both Cx46 and Cx43 (A) or only for Cx46 (B) or Cx43 (C) vs. the total number of cells when cultured under normoxic conditions or for 12 days in hypoxic conditions. Bars represent the values obtained in 3 independent experiments. Each value (represented by black circles) corresponds to the average of the values obtained in 7 different images from the same experiment. *Asterisks* indicate a significant difference in the values between the different incubation conditions ($p<0.05$).
